# The Possible Role of Adipokines in HCV Associated Hepatocellular Carcinoma

**DOI:** 10.31557/APJCP.2020.21.3.599

**Published:** 2020-03

**Authors:** Usama M El-Daly, Magdy M Saber, Mona S Abdellateif, Hanan R Nassar, Alfred E Namour, Yahia M Ismail, Abdel-Rahman N Zekri

**Affiliations:** 1 *Department of Medical Oncology, Damietta Oncology Center, Damietta, *; 2 *Department of Medical Oncology and Malignant Hematology, *; 3 *Medical Biochemistry and Molecular Biology, Cancer Biology Department, *; 4 *Molecular Virology and Immunology Unit, Department of Cancer Biology, National Cancer Institute, Cairo University, Cairo, Egypt. *

**Keywords:** Adiponectin, leptin, visfatin, adipokines, HCC, HCV

## Abstract

**Background:**

Adipokines play an important role in the regulation of inflammation and tumor progression.

**Aim:**

Assessment of the possible role of adiponectin, leptin and visfatin in HCV associated hepatocellular carcinoma (HCC).

**Methods:**

patients were classified into 85 patients with HCV associated HCC, 100 patients with chronic hepatitis C viral (HCV) infection compared to 50 normal control (NC) subjects. All subjects included in the study were assessed for HCV infection by seropositive HCV antibodies, as well as HCV RNA by RT-PCR. Serum levels of adiponectin, leptin and visfatin were assessed using enzyme linked immunosorbent assay (ELISA). The data were correlated to the relevant clinic-pathological features of the patients, and the overall survival (OS) rate.

**Results:**

There was a significant difference in the serum levels of adiponectin and visfatin among HCC, HCV and NC groups (P<0.001). The serum levels of leptin and alpha fetoprotein (AFP) were significantly higher in HCC group (P<0.001). There was a significant association between the serum level of adiponectin and advanced Child class liver cirrhosis (P=0.03), as well as with poor performance status (ECOG, P=0.02). Serum leptin associated significantly with the number of lesions in the liver (P=0.006), visfatin associated with increased mortality rate (P<0.001). Adiponectin, leptin and visfatin associated significantly with liver cirrhosis in HCV patients (P<0.01). Leptin achieved the highest sensitivity (98.8%). visfatin achieved the highest specificity (100%) and PPV (100%) for detection of HCC. The combination of serum leptin and visfatin for the diagnosis of HCV associated HCC showed sensitivity, specificity, PPV, NPV and accuracy (100%, 96.6%, 93.4%, 100% and 97.4%; respectively).

**Conclusion:**

Adiponectin, leptin and visfatin have an important role(s) in the pathogenesis of HCV associated HCC.

## Introduction

Hepatocellular carcinoma (HCC) is the third most common cause of cancer related death worldwide (Siegel et al., 2017). In Egypt, HCC ranks the first among cancers in males (33.6%), and the 2nd in females after breast cancer (13.5%), with HCV genotype 4 is the most prevalent underlying cause (Zekri et al., 2001; Ibrahim et al., 2014). The most commonly aberrant gene expression in Egyptian HCC patients are related to virus response such as; ISGF3G, virus-induced signaling adapter VISA and FGR (Zekri et al., 2012). The non-alcoholic fatty liver disease (NAFLD) is considered an important cause of end stage liver disease and HCC in the developed countries with a worldwide prevalence ranging from 6% to 35% (Said and Ghufran, 2017). 

Adipose tissue has an important role in the pathogenesis of hepatic diseases, since it secretes pro- and anti-inflammatory cytokines called adipokines which include e.g. adipocytokines, leptin, visfatin, resistin, chemerin, retinol-binding protein 4 and irisin (Boutari et al., 2018). Leptin, visfatin and adiponectin play a role in the regulation of inflammation, tumor microenvironment and progression of hepatic diseses (Deng et al., 2016). Changes in these adipokines levels are associated with increased risks of breast (Macis et al., 2014; Ye et al., 2014), colorectal (Zekri et al., 2015; Otani et al., 2017), liver (Song and Gu, 2015) and pancreatic cancers (Stolzenberg-Solomon et al., 2015).

Adiponectin is a hormone released by the adipose tissue, which improves the hepatic and peripheral insulin resistance (IR). It has anti-inflammatory and hepato-protective activities (Polyzos et al., 2010), as it stimulates the secretion of anti-inflammatory cytokines such as interleukin-10 (IL-10) and IL-1 receptor antagonist. It also suppresses the release of pro-inflammatory cytokines such tumor necrosis factor α (TNF-α), IL-6 and interferon-γ. this will Consequently lead to blocking the activation of the nuclear factor κB (Boutari et al., 2018). 

Leptin is expressed mainly in adipose tissue, and it is involved in the regulation of energy homeostasis, angiogenesis, hematopoiesis and neuroendocrine function (Matarese et al., 2010). In the liver, it decreases the expression of sterol regulatory element-binding transcription factor 1 (SREBP-1) (Kakuma et al., 2000), which regulates the expression of genes required for glucose metabolism, fatty acid, and lipid production (Ferre and Foufelle, 2010). In addition, it has a key-role in hepatic fibrogenesis by up-regulating the expression level of the transforming growth factor β1, with subsequent activation of stellate cells, and increasing the fibrogenic response in the liver (Ikejima et al., 2001).

Visfatin is another cytokine which is secreted by the adipose tissue. It is called pre-B cell colony-enhancing factor, and it is a pro-inflammatory cytokine that stimulates TNF-α and IL-6 secretion (Moschen et al., 2007). It is also involved in the development of NAFLD by regulating hepatic inflammation as well as glucose homeostasis and IR (Saxen and Anania, 2015).

Therefore, we assessed serum levels of adiponectin, leptin and visfatin in HCC and chronic HCV patients compared to healthy individuals matched for age and sex as control. We also evaluated their relation with the survival rates of patients. In addition, correlations between these markers and the relevant clinic-pathological features of the patients.

## Materials and Methods

*Patients*

This retrospective cohort study included 85 patients with histo-pathologically confirmed HCC on top of hepatitis C virus (HCV), and 100 patients with chronic HCV, compared to 50 matched normal controls (NC). Patients were diagnosed and treated at the National Cancer Institute (NCI), Kasr Al Ainy faculty of medicine, Cairo University, and from Damietta Oncology Centre outpatient clinics, during the period between October 2012 and January 2014. The NC subjects had normal serum ALT, AST levels and all were seronegative for HBV and HCV antibodies. 

The HCC and HCV patients were HCV/ genotype 4 positive by seropositive HCV antibodies (EIAgen HCV Ab (v.4), code: 071064, adaltis. Milano Italy) as well as, HCV RNA by RT-PCR (artus HCV RT-PCR Kits CE, 4518265, QIAGEN GmbH, QIAGEN Strasse 1, D-40724 Hilden). Genotyping was done through amplification of the biotinylated complimentary DNA (cDNA) product of the core region of the HCV RNA using the HCV Amplification 2.0 kit (LiPA; Bayer HealthCare, Tarrytown, NY, USA). Then the product was processed using the VERSANT HCV Genotype 2.0 assay (LiPA; Bayer HealthCare, Eragny, France), according to the manufacturer’s instructions (Stuyver et al., 1996).

All patients were subjected to full history taking, clinical and radiological examination, as well as serological examination including AST, ALT, total and direct bilirubin, serum albumin-prothrombin time and INR), Complete blood picture, Kidney function tests, Random blood sugar and alpha feto-protein. Patients were confirmed for HCC daignosis by abdominal ultrasonography and triphasic CT abdomen, they had not received chemotherapy, targeted therapy or radiotherapy, also they had not undergone hepatic resection or any local ablative therapy like RFA, PEI or TACE. 

Patients were excluded from the study if they had; 1) causes of liver disease other than HCV, 2) Hepatic viral infection other than HCV and 3) Patients with other malignancies. 

Body mass index (BMI) was calculated by dividing the body weight in kilograms by the square of the patient’s height in meters (WHO, Fact sheet No 164, 2016).

Histopathological diagnosis and grading were performed according to classification criteria of the World Health Organization (WHO/HIV/2016), and staging was done according to the American Joint Committee on Cancer (Amin et al., 2017). 

*Assessment of Adipocytokines concentrations in serum samples*

Serum sample collection: Blood samples were collected in serum separator tubes (SST) and allowed to clot for 10 to 20 minutes at room temperature. Samples were then centrifuged at 1,000 g for 10 minutes, and the serum was aliquot and stored at -20°C until use for protein evaluations.

Serum levels of Adiponectin, leptin and vesfatin were assessed using the enzyme linked immunosorbent assay (ELISA), according to manufacturer’s instructions (Human Adiponectin Platinum ELISA Affymetrixe Bioscience), Human Leptin (LEP) ELISA Kit WKEA MED SUPPLIES and RayBio Human Visfatin Enzyme Immunoassay Kit RayBiotech; respectively). The tests were performed using a microplate reader (Tecan) at 450 nm wavelength.

*Statistical methods*

The data were analyzed using SPSS version 22 and graph pad prism 7. Non-parametric Mann-Whitney and Kruskal-Wallis test were used to compare markers among groups, and its association to clinic-pathological variables. Patients and their tumor characteristics were analyzed using Chi-square test. The Area under the receiver operating curve (ROC) was calculated for each marker to investigate the best cut-off level for diagnosis of HCC. The association with overall survival was analyzed by log-rank test and Cox-regression for multivariate analysis. All p-values were considered statistically significant at <0.05

## Results

*Patients’ characteristics*

The mean ages of the HCC, chronic HCV and control groups were (60.7, 58 and 57; respectively). The mean weight was (75.8, 79.3 and 76.7 Kg; respectively), and the BMI was (27.2, 28.4 and 28.6; respectively, Table 1).

Out of the 85 patients in HCC group, there were 68(80%) males, and 17(20%) females. ECOG performance status was 0 in 16 (18.8%) of patients, ECOG 1 in 22 (25.9%), ECOG 2 in 34 (40.0%), and ECOG 3 in 13 (15.3%) patients. Twenty-seven (31.8%) patients were normal weight, and 38 (44.7%) were overweight, 14 (16.5%) were class I obesity, and 6 (7.1%) were class II obesity. Fourteen patients (16.5%) were diabetic, and 71(83.5%) were non diabetic. All patients had liver cirrhosis. Twenty patients (23.5%) were score A Child-Turcotte-Pugh, 45 (52.9%) were score B and 20 patients (23.5%) were score C. Nineteen patients (22.4%) had tumor size less than 5 cm, meanwhile 66 (77.6%) patients had tumor size more than 5 cm. Barcelona Clinic Liver Cancer (BCLC) stage was A in 3 (3.5%), B in 27 (31.8%), C in 43 (50.6%), and D in 12 (14.1%) patients. thirty-five (41.2%) patients had HCC in the right lobe, 18 (21.2%) in the left lobe, and 32 (37.6%) in both lobes. Twenty patients (23.5%) had single lesion, six (7.1%) had bifocal lesions, and 59 (69.4%) had multiple lesions. Distant metastasis was present in 14 (16.5%) patients only. At the end of the study 15 (17.6%) patients were alive, and 70 (82.4%) were dead (Table 2).

*Serum levels of the studied markers in the patients’ groups*

**Figure 1:**
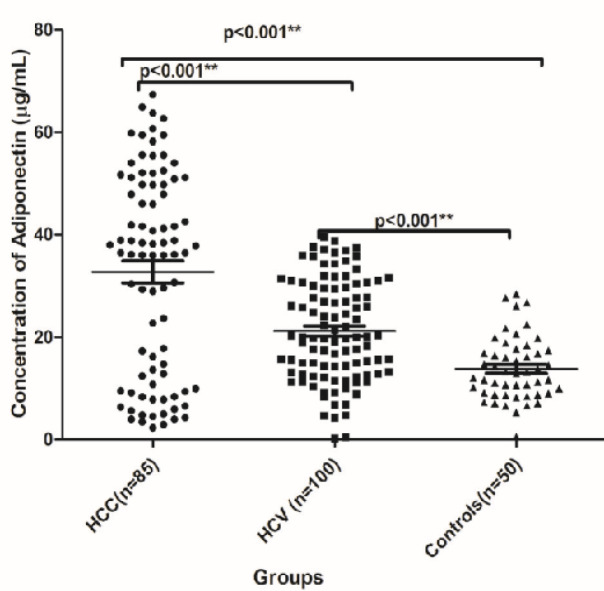
The Serum Level of Adiponectin (µg/ml) among HCC, HCV and NC Groups

**Figure 2:**
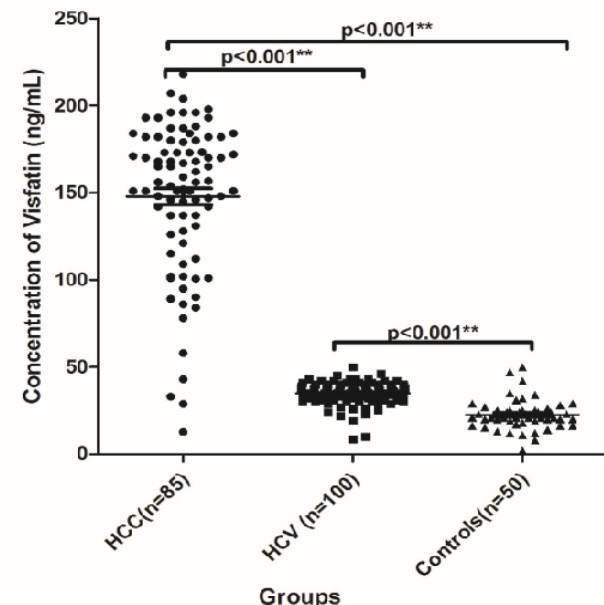
The Serum Level of Visfatin (ng/ml) among HCC, HCV and NC Groups

**Figure 3:**
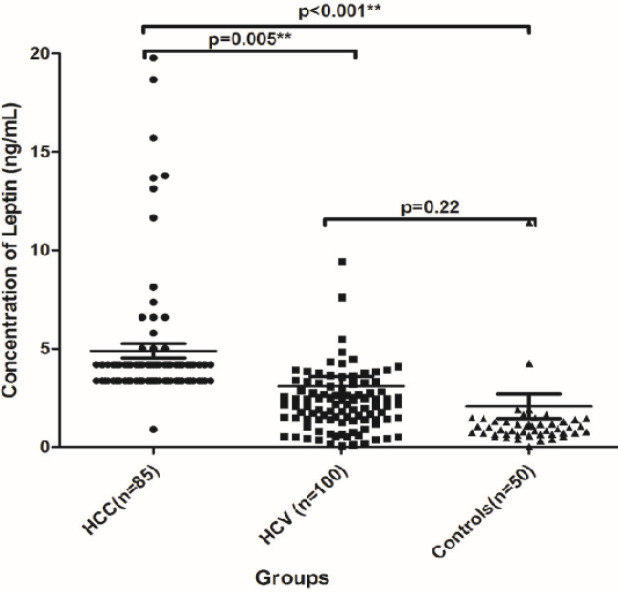
The Serum Level of Leptin (µg/ml) among HCC, HCV and NC Groups

There was a significant difference in the serum levels of adiponectin (median µg/ml and range) among HCC, HCV and NC groups; 36.5 (2.3- 67.4), 20.1 (0.30- 39.6) and 13.1 (28.4- 553.2); respectively, (P<0.001, Figure 1). Also there was significant difference in the serum level of visfatin (median ng/ml and range) among HCC, HCV and NC groups; 156.0 (12.7- 218), 35.0 (8.5- 50) and 21.0 (2.0- 50); respectively, (P<0.001, Figure 2). There was a significant increase in the serum level of leptin in HCC group which was 4.19 ng/ml (0.9- 19.8, P<0.001), whereas there was no significant difference in its level between HCV and the NC groups; it was 2.2 ng/ml (0.07- 34.0) versus 0.99 ng/ml (0.02- 22.0); respectively (P=0.22, Figure 3). AFP was also significantly increased in the HCC group as it was 272.0 ng/ml (1.9- 11884, P<0.001) compared to the other groups. However, no significant difference was found in its level between the HCV and the NC groups where its level was 7.5 (1.0- 146) and 5.4 (2.6-9.6) ng/ml; respectively (P<0.05, Table 3).

*Correlation between the studied markers and the clinico-pathological features of the HCC patients*

There was significant association between increased serum level of adiponectin and advanced Child class liver cirrhosis (P=0.03), and poor performance status (ECOG) of the patients (P=0.02). Leptin associated significantly with the number of hepatic lesions (P=0.006), Whereas increased serum level of visfatin associated significantly with the increased mortality of the patients (P<0.001). No significant relations were found between the serum levels of adiponectin, leptin, visfatin and the age of the patients (P=0.61, 0.89 and 0.50; respectively), residence (P=0.16, 0.97 and 0.28; respectively), BMI (P=0.69, 0.63 and 0.68; respectively), the presence of ascites (P=0.33, 0.32 and 0.61; respectively), BCLC (P=0.38, 0.22 and 0.77; respectively), tumor size (P=0.21, 0.30 and 0.14; respectively), metastasis (P=0.66, 0.39 and 0.20; respectively), or the presence of diabetes (P=0.44, 0.78 and 0.99; respectively, Table 4).

*Correlation between the studied markers and the clinico-pathological features of HCV patients*

Serum levels of adiponectin, leptin and visfatin associated significantly with the presence of cirrhosis in HCV patients (P<0.001, P= 0.008, P <0.001; respectively). Also serum levels of adiponectin and visfatin associated significantly with the age of the patients. However, there were no significant association with other features like BMI and DM (P>0.05, Table 5).

*The diagnostic accuracy of serum adipokines and AFP for HCC*

**Figure 4:**
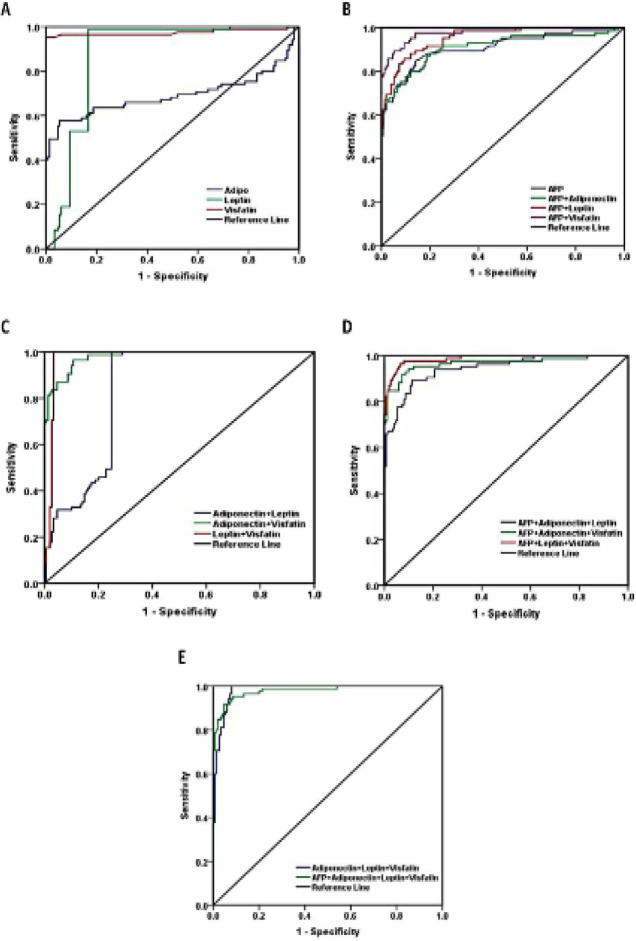
ROC Curve Analysis of the Serum Adiponectin, Leptin, Visfatin and AFP for the Diagnosis of HCC Patients

To evaluate the diagnostic accuracy of serum adipokines in HCC patients, HCV and NC subjects, ROC curve analysis was performed. The AUC of serum adiponectin was 0.691 (95%CI=0.87-0.95), the sensitivity, specificity, PPV, NPV and accuracy were (88.2%, 80.7%, 72.1%, 92.4% and 83.4%; respectively). The AUC of serum leptin was 0.875 (95%CI=0.83-0.92). The sensitivity, specificity, PPV, NPV and accuracy were (98.8%, 83.3%, 77.1%, 99.2%and 88.9%; respectively), where leptin achieved the highest sensitivity (98.8%). While the AUC of the serum visfatin was 0.975 (95%CI=0.95-1.0). The sensitivity, specificity, PPV, NPV and accuracy were (95.3%, 100%, 100%, 97.4% and 98.3%; respectively), where visfatin had the highest specificity and PPV (100%). On the other hand, the AUC of the serum AFP was 0.910 (95%CI=0.87-0.95). whereas the sensitivity, specificity, PPV, NPV and accuracy were (88.2%, 80.7%, 72.1%, 92.4% and 83.4%; respectively, Table 6 and Figure 4).

By performing different combinations between adiponectin, leptin, visfatin and AFP for diagnosis of HCC patients. We found that leptin and visfatin combination achieved the highest sensitivity, specificity, PPV, NPV and accuracy (100%, 96.6%, 93.4%, 100% and 97.4%; respectively, Table 6). 

*Overall survival analysis in HCC patients*

**Figure 5:**
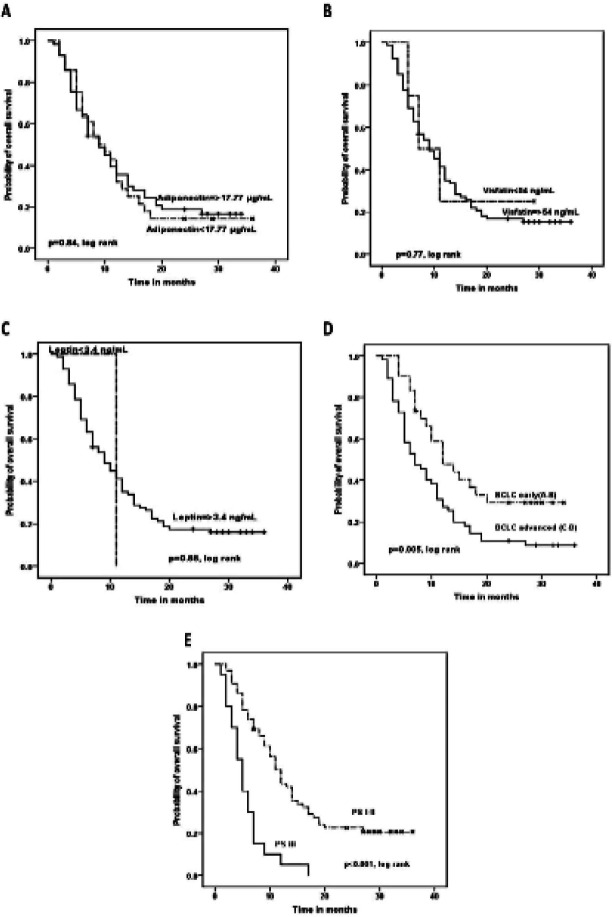
Kaplan Meier Aanalysis for the Overall Survival (OS) of HCC Patients

Kaplan Meier analysis for overall survival (OS) of HCC patients was done. PS of the patients associated significantly with shorter OS. The median OS of patients with PS I and II was 11 months versus 5 months for patients with PS III (P<0.001). Also advanced BCLC class associated significantly with shorter OS, where the median OS of patients with BCLC (A and B) versus those with advanced BCLC (C and D) were (12 and 7; respectively). On contrary, there were no significant impact of serum adipokine levels or AFP on the OS of the patients (Table 7 and Figure 5). 

Multivariate analysis for OS showed that only BCLC is an independent prognostic factor for OS (Table 8). 

**Table 1:** Baseline Characteristics of the 3 Studied Groups

Variable	HCC (85)	HCV (100)	Normal control (50)	p-value
Age (years)	60.7±8.8*	58.1±8.6	57.7±8.2	0.17
Weight	75.8±14.4	79.3±13.3	76.7±16.8	P=0.26^x^
Height (cm)	166.71 ±8.5	164.33 ±8.7	165.20 ±15.6	0.13
BMI	27.2±4.95	28.4±4.1	28.6±6.6	P=0.21^x^
Diabetes mellitus	14 (16.5%)	30 (30.0%)	16 (32.0%)	P=0.054^y^
HGB	12.2±1.9^a^	13.3±1.6^b^	14.1±1.3^c^	P<0.001^x^
WBCs	6.3±4.7	5.6±2.0	6.8±1.5	P=0.07^x^
Platelets	135.3±72.7^a^	171.5±71.17^b^	306.2±82.3^c^	P<0.001
Cirrhosis	85(100.0)	48(48.0)	NA	P<0.001^y^
Albumin	2.96±0.59	3.8±0.63	NA	P<0.001^z^
ALT	63.9±35.5^a^	59.9±47.5^b^	12.5±4.3^c^	P<0.001^x^
AST	89.7±44.1^a^	63.3±47.7^b^	21.7±6.2^c^	P<0.001^x^
INF	64.0±13.3	86.6±17.8	NA	P<0.001^z^
Bilirubin	2.2±2.4	0.78±0.3	NA	P<0.001^z^

*, Data are expressed as mean±SD; Groups bearing different initials are significantly different; x, Kruskal-Wallis test; y, Chi square test; z, Mann-Whitney test

**Table 2:** Clinico-Pathological Features of HCC Patients

Parameter (85)	Number (%)
Age	60.7±8.8 (43-84)
Gender	
Male	68 (80)
Female	17 (20)
Residence	
Urban	24 (28.2)
Rural	61 (71.8)
Performance status (ECOG)	
0	16 (18.8)
1	22 (25.9)
2	34 (40.0)
3	13 (15.3)
BMI category	
Normal Weight	27 (31.8)
Overweight	38 (44.7)
Class I obesity	14 (16.5)
Class II obesity	6 (7.1)
Diabetes Mellitus (DM)	
Yes	14 (16.5)
No	71 (83.5)
Cirrhosis	
Yes	0 (0.0)
No	85 (100.0)
Schistosomiasis	
Yes	51 (60%)
No	43 (40%)
Ascites	
Yes	31 (36.5%)
No	54 (63.5%)
Child-Turcotte-Pugh score	
A	20 (23.5%)
B	45 (52.9%)
C	20 (23.5%)
Tumor size (cm)	
≤5 cm	19 (22.4)
>5 cm	66 (77.6)
Affected lobe	
Right	35 (41.2%)
Left	18 (21.2%)
Both	32 (37.6%)
Number of lesions	
Single	20 (23.5%)
Bifocal	6 (7.1%)
Multiple	59 (69.4%)
Metastasis	
M0	71 (83.5%)
M1	14 (16.5%)
Parameter (85)	Number (%)
BCLC	
A	3 (3.5)
B	27 (31.8)
C	43 (50.6)
D	12 (14.1)
Mortality	
Alive	15 (17.6)
Dead	70 (82.4)

**Table 3:** Serum Levels of the Studied Markers among the Different Investigated Groups

Marker	HCC (85)	HCV (100)	NC (50)	p-value
Adiponectin				
Median (µg/ml)	36.5 ^a^	20.1 ^b^	13.1 ^c^	
Range	2.3- 67.4	0.30- 39.6	28.4- 553.2	<0.001
IQR	39.9	16.9	8.53	
95% CI	28.5- 37.1	19.2- 23.1	12.1- 15.6	
Leptin				
Median (ng/ml)	4.19 ^a^	2.2 ^b^	0.99 ^b^	<0.001
Range	0.9- 19.8	0.07- 34.0	0.02- 22.0	
IQR	0.82	1.85	0.76	
95% CI	4.2- 5.6	2.1-4.1	0.8-3.3	
Visfatin				
Median (ng/ml)	156.0 ^a^	35.0 ^b^	21.0 ^c^	<0.001
Range	12.7- 218	8.5- 50	2.0- 50	
IQR	53	7	7.5	
95% CI	138.5-157.0	33.7-36.3	20.1-25.1	
AFP				
Median (ng/ml)	272.0 ^a^	7.5 ^b^	5.4 ^b^	
Range	1.9- 11884	1.0- 146	2.6-9.6	<0.001
IQR	1173	9	3.45	
95% CI	1414.0- 9315.7	10.1- 18.6	5.0- 6.1	

**Table 4:** Correlation between the Studied Markers and the Clinico-Pathological Features of the HCC Patients

Characteristics	Adiponectin	p	Leptin	p	Visfatin	p
Age						
Age<60 (n=41)	38.4 (2.3-64.99)	0.61^a^	4.2 (3.4-15.7)	0.89^a^	156 (78-207)	0.50^a^
Age≥60 (n=44)	36.1 (2.95-67.4)		4.2 (0.9-19.8)		155.5 (12.7-218.0)	
Residence						
Rural (n=61)	38.03 (3.99-67.4)	0.16^a^	4.2 (3.4-19.8)	0.97^a^	154 (12.7-218)	0.28^a^
Urban (n=24)	33.6 (2.3-62.7)		4.2 (0.9-13.8)		166.5 (28.8-198)	
DM						
Non Diabetic (n=71)	36.2 (2.96-62.7)	0.44^a^	4.2 (0.9-19.8)	0.78^a^	156 (28.8-218)	0.99^a^
Diabetic (n=14)	44.1 (2.3-67.4)		3.8 (3.4-15.7)		160.5 (12.7-204)	
BMI						
Normal (n=27)	29.6 (3.4-62.7)	0.69^b^	3.4 (3.4-7.4)	0.63^b^	162 (12.7- 207)	0.68^b^
Overweight (n=38)	36.1 (2.96-67.41)		4.2 (0.9- 19.8)		156 (28.8- 204)	
Class I obesity (n=14)	40.4 (2.3-59.5)		4.2 (4.3.8-18.7)		160.5 (33- 193)	
Class II obesity (n=6)	44.5 (4.02-55.5)		3.8 (3.4-15.7)		126 (43- 218)	
Ascites						
No (n=54)	36.1 (2.3- 67.4)	0.33^a^	3.8 (3.4-19.8)	0.32^a^	155 (12.8- 218)	0.61^a^
Yes (n=31)	38.4 (2.96-64.99)		4.2 (0.9-18.7)		156.8 (78- 204)	
Child-class						
I (n=20)	14.2 (2.3-59.5)	0.03*^b^	3.4 (3.4-6.6)	0.53^b^	170 (43-193)	0.62^b^
II (n=45)	40.8 (2.96- 67.4)		4.2 (0.9- 19.8)		151 (12.8- 218)	
III (n=20)	38.4 (4.3- 62.7)		3.8 (3.4- 13.1)		157.9 (33- 207)	
Performance status (ECOG)						
0 (n=16)	14.4 (4.02-58.3)	0.02*^b^	3.8 (3.4-8.1)	0.74^b^	168.5 (43.0-193.0)	0.85^b^
1 (n=22)	37.2 (2.3-63.8)		3.8 (0.9-19.8)		152.5 (89.0-193.0)	
2 (n=34)	38.6 (2.9- 67.4)		4.2 (3.8-18.7)		154 (12.7-198.0)	
3 (n=13)	49.8 (9.5 -64.9)		4.2 (3.4-13.7)		159 (95- 218)	
Number of lesions						
Single (n=20)	29.3 (2.3- 51.7)	0.11^b^	4.2 (3.2-18.7)	0.006*^b^	163.4 (12.7-187)	0.70^b^
Bifocal (n=6)	33.3 (7.8-55.5)		6.2 (4.2-15.7)		145.5 (100.6-180)	
Multiple (n=59)	38.9 (3.4-67.4)		3.4 (0.9-19.8)		156 (33- 218)	
BCLC						
A (n=3)	35.9 (5.0- 36.03)	0.38^b^	4.2 (4.2-4.2)	0.22^b^	90 (86- 182)	0.77^b^
B (n=27)	37.8 (4.02-63.8)		3.4 (3.4-15.7)		152 (43- 198)	
C (n=43)	36.2 (2.3- 67.4)		4.2 (0.9-19.8)		165 (12.7- 218)	
D (n=12)	40.1 (4.8-64.9		4.2 (3.4- 13.7)		152 (95- 207)	
Tumor size(cm)						
< 3 (n=8)	36.9 (6.6- 55.5)	0.21^b^	4.2 (3.4- 15.7)	0.30^b^	139.5 (58- 182)	0.14^b^
3-5 (n=11)	28.9 (2.3- 47.9)		4.2 (3.4- 8.1)		156 (28.8- 184)	
> 5 (n=66)	38.3 (2.9-67.4)		3.4 (0.9-19.8)		163.5 (12.7-218.0)	
Metastasis						
M0 (n=71)	36.5 (2.3-67.4)	0.66^a^	4.2 (3.4- 18.7)	0.39^a^	152 (28.8- 207)	0.20^a^
M1 (n=14)	37.3 (7.8- 64.9)		3.4 (0.9- 19.8)		166.5 (12.7- 218)	
Mortality						
Alive (n=15)	36.5 (4.02-60.75)	0.73^a^	4.2 (3.4- 15.7)	0.64^a^	109 (43- 182)	<0.001*^a^
Dead (n=70)	37.2 (2.3-67.4)		3.8 (0.9-19.8)		165 (12.7-218.0)	

^a^, Mann whitney test; ^b^, Kruskal Wallis test; *, Significance at p<0.05

**Table 5:** Correlation between the Studied Markers and the Clinico-Pathological Features of the HCV Patients

Parameters	Adiponectin	p	Leptin	p	Visfatin		AFP	P
Age		0.015^a^		0.83		0.008		0.98
<60 (73)	17.7 (0.3-37.5)		2.4 (0.07-27.9)		35 (8.5-43.0)		8 (1-146)	
≥60 (27)	27 (0.5-39.6)		2.1 (0.5-34.0)		38 (29-50)		6 (1-77)	
Gender		0.15^a^		0.24		0.53		0.75
Male (69)	19.7 (4.2-39.6)		2 (0.07-34.0)		36 (8.5-45)		7 (1-146)	
Female (31)	25.8 (0.3-37.5)		2.4 (0.4-7.6)		35 (10-50)		8 (1-87)	
DM		0.19^a^		0.5		0.64		0.52
Nondiabetic (70)	19.8 (0.3-39.6)		2.3 (0.07-27.9)		35 (8.5-46)		8 (1-87)	
Diabetic (30)	26.5 (0.5-37.0)		1.9 (0.4-34.0)		36.5 (10-50)		6 (1-146)	
BMI		0.48^b^		0.8		0.94		0.64
Normal (20)	15 (4.2-39.6)		2.2 (0.4-9.4)		35 (19-43)		6 (1-55)	
Overweight(50)	20.1 (0.5-37.6)		2.1 (0.07-34.0)		35.5 (8.5-50)		8 (2-62)	
Class I Obesity (25)	20.2 (0.3-38.9)		2.2 (0.15-5.5)		36 (10-43)		6 (1-146)	
Class II Obesity (5)	24.1 (13.2-35.9)		3 (0.4-3.4)		33 (25-45)		9 (3-77)	
Cirrhosis		<0.001^a^		0.008		<0.001		0.3
Absent (52)	14.7 (0.3-34.3)		1.7 (0.07-4.8)		34 (8.5-43)		5.5 (1-87)	
Present (48)	29.6 (10.1-39.6)		2.4 (1.2-34.0)		38 (26-50)		9 (1-146)	

^a^, Mann whitney,^ b,^ Kruskal wallis

**Table 6:** The Diagnostic Accuracy of ROC-Curve Analysis for the Different Studied Markers

	Cutoff	AUC	SE (95%CI)	Sensitivity	Specificity	PPV	NPV	Accuracy
AFP	10.5	0.91	0.022 (0.87-0.95)	88.2	80.7	72.1	92.4	83.4
Adiponectin	17.8	0.691	0.044 (0.60-0.78)	67.1	54.7	45.6	74.5	59.1
Leptin	3.36	0.875	0.024 (0.83-0.92)	98.8	83.3	77.1	99.2	88.9
visfatin	54	0.975	0.015 (0.95-1.0)	95.3	100	100	97.4	98.3
Adiponectin+leptin	0.4667	0.838	0.026 (0.79-0.89)	100	75.2	69.7	100	83.8
Adiponectin+ visfatin	0.6407	0.98	0.007 (0.97-0.99)	94.1	90	84.2	96.4	91.5
leptin+visfatin	0.351	0.976	0.011 (0.95-1.0)	100	96.6	93.4	100	97.4
Adiponectin+leptin+visfatin	0.4257	0.982	0.007 (0.97-1.0)	100	92	87.6	100	94.9
AFP+ Adiponectin	0.1616	0.91	0.023 (0.87-0.95)	88.2	80	71.4	92.3	83
AFP+ leptin	0.157	0.954	0.012 (0.93-0.98)	89.4	86	78.4	93.5	96.2
AFP+ visfatin	0.1218	0.977	0.009 (0.96-1.0)	92.7	90.7	84.9	95.8	91.5
AFP+ adiponectin+leptin	0.16139	0.945	0.015 (0.92-0.97)	89.4	88.7	81.7	93.7	88.9
AFP+ adiponectin+visfatin	0.1338	0.967	0.013 (0.94-0.99)	92.9	91.3	85.9	95.8	91.9
AFP+ leptin+visfatin	0.10249	0.987	0.006 (0.98-1.0)	96.5	93.3	89.1	97.9	94.5
AFP+ adiponectin+leptin+visfatin	0.1028	0.98	0.008 (0.96-1.0)	95.3	91.3	86.2	97.2	92.8

**Table 7:** Overall Survival Analysis for HCC Patients

Factor	No	OS			P
		%	Median	95% CI	
Total		17.6	NA	NA	NA
Age					0.52
<60	41	19.5	10	7.0-13.0	
≥60	44	15.9	7	4.2-9.8	
Gender					0.19
Male	68	14.7	9	6.6-11.4	
Female	17	29.4	14	5.2-22.7	
Residence					0.26
Rural	61	19.7	11	8.3-13.7	
Urban	24	12.5	6	3.9-8.1	
BMI					0.37
Normal	27	18.5	7	2.0-12.0	
Overweight-Obese	58	17.2	10	6.8-13.2	
PS					<0.001*
I-II	65	23.1	11	9.1-12.9	
III	20	0	5	3.6-6.4	
Tumor size (cm)					0.15
≤5	19	26.3	12	5.2-18.8	
>5	66	15.2	8	5.6-10.3	
Child class					0.09
A	54	20.4	11	8.4-13.6	
B	31	12.9	6	4.0-8.0	
BCLC					0.005*
Early (A-B)	30	33.3	12	6.9-17.0	
Advanced (C-D)	55	9.1	7	4.1-9.9	
Lobe					0.55
1	20	20	10	5.6-14.4	
2-3	65	16.9	9	6.2-11.8	
Metastasis					0.34
M0	71	19.7	10	7.4-12.6	
M1	14	7.1	7	5.2-8.8	
AFP					0.22
<10.5	10	30	12	7.4-16.6	
≥10.5	75	16	9	6.8-11.2	
Adiponectin (/ml)					0.84
<17.77	28	14.3	9	4.9-13.1	
≥17.77	57	19.3	9	6.3-11.7	
Leptin (ng/ml)					0.88
<3.36	1	0	11	NA	
≥3.36	84	17.9	9	6.2-11.9	
Visfatin (ng/ml)					0.77
<54.0	4	25	7	1.1-12.9	
≥54.0	81	17.3	9	6.2-11.8	

**Table 8:** Multivariate Survival Analysis for HCC Patients

Factors	Overall survival		
	HR	95% CI	P-value^a^
PS III vs I-II	1.61	0.93-2.80	0.09
BCLC C-D vs A-B	2.96	1.7-5.3	<0.001*

^a^, Cox regression; *Significance at p<0.05

## Discussion

The NAFLD associated HCC incidence is increasing worldwide in parallel with obesity epidemic (Said and Ghufran, 2017). However, few studies investigated the adipokines levels and HCC risk among patients with HBV and/or HCV (Chen et al., 2014; Tsai et al., 2017). In the corresponding study, we evaluated the prognostic and the predictive roles of adiponectin, leptin and visfatin in HCV associated HCC patients, chronic HCV patients and normal control subjects, with a specific aim of identifying different pathways that may be implicated in the pathogenesis of HCV associated HCC.

Till now, there is a discrepancy in the published data regarding the level of adiponectin in HCC patients. Our results demonstrate a significant increase in the serum level of adiponectin in HCC and HCV patients compared to the NC groups. We also found a significant association between the elevated serum level of adiponectin and advanced Child class liver cirrhosis as well as with poor ECOG performance status of the tested patients. These data are in agreement with the other previously published data in the literature reported that higher adiponectin level associated significantly with increased risk of HCC (Chen et al., 2014; Song and Gu, 2015), advanced liver disease (Kaser et al., 2005; Liu et al., 2009) and predicts worse patients’ prognosis (Wang et al., 2014; Siegel et al., 2015;). Similarly, Chen et al., (2012) performed a study on HCC patients’ tissues, he found that increased adiponectin level correlated significantly with tumor size, indicating its possible role as a prognostic factor in those patients. In contrast to the previously mentioned studies, Shin et al., (2014) reported a significant relationship between adiponectin and favorable prognosis in HCC patients. Similarly, Starley et al., (2010) and Bråkenhielm et al., (2004) reported decreased amount of adiponectin in HCC patients. They demonstrated in their studies that adiponectin inhibits angiogenesis and primary tumor growth through inhibition of neovascularization and enhancement of tumor cell apoptosis (Shin et al., 2014; Wong et al., 2016). Although the molecular and biological mechanisms explaining the association between increased adiponectin and reduced survival in HCC patients are not clearly understood, possible explanations for this association includes reduced liver function e.g. elevated liver enzymes (Wang et al., 2014), since the adiponectin level reflects the severity of liver fibrosis (Liu et al., 2009) and cirrhosis (Kaser et al., 2005) which are correlated with consequent poorer prognosis.

Our data also show a significant increase in the serum levels of visfatin in HCC and HCV patients compared to the NC groups. These data are in concordance with these of Ninomiya et al., (2011) and Liang et al., (2018) who found that visfatin level is significantly higher in HCC patients compared to control group, and that it induces HCC cell migration via upregulation of miR-21. In our study, we found that increased serum level of visfatin associates significantly with increased mortality rates of the patients. These results are in concordance with that reported by Tsai et al., (2017) who found that visfatin level correlates significantly with tumor size, LN metastasis and shorter survival time. Hence it could be considered a poor prognostic marker of HCC patients. 

Regarding the serum level of leptin, our data demonstrated a significant increase in its level in HCC patients compared to the other tested groups, whereas there were no significant differences in its level between HCV and NC. These data are in agreement with those reported by Watanabe et al., (2011) that leptin can cause tumor growth, and associates with HCC recurrence after treatment. Since it has an important role in carcinogenesis, cancer cell survival, proliferation and migration (Booth et al., 2015). It also increases the expression levels of anti-apoptotic proteins, inflammatory markers (TNF-a, IL-6), angiogenic factors (VEGF), and the hypoxia-inducible factor-1a (HIF-1a) (Sharma et al., 2006). This was correlated with our finding that Leptin overexpression was significantly associated with increased number of the lesions in the liver. However, these data are not in agreement with Chen et al., (2014) who conducted a prospective study in Taiwan, and reported no significant association between the serum levels of leptin or visfatin and increased risk of HCC.

The overall survival analysis of HCC patients showed that only PS and BCLC associate significantly with OS. Meanwhile, no significant impact is present for serum adiponectine, leptin, visfatin or AFP on the OS of the patients. These results are in concordance with those of Shen et al., (2016), and in contrast with those of Sun et al., (2017) who reported that HCC patients with high serum visfatin levels had significantly shorter OS times compared to those with low serum visfatin levels.

Many previous studies addressed the role of adipokines and obesity in the pathogenesis of NAFLD and HCC (Noureddin and Rinella, 2015; Perumpail et al., 2015), however none of these investigate their role in HCV chronic hepatitis and its progression to HCC. In the current study we investigated this point, we found a significant increase in the serum levels of adiponecine, leptin and visfatin in HCV chronic hepatitis patients compared to normal control. Our data in this context confirm the data of Tsai et al., (2017), who reported increased serum visfatin level in HCC patients with HBV or HCV infection. It was demonstrated that hepatic steatosis, along with obesity and diabetes mellitus (DM), increase the risk of HCC in patients with chronic HCV (Nam, 2017). Our data show significant differences between those three studied groups regarding DM, since 30% of the patients had DM in the HCV group, compared to 16.5% in HCC, and 32% in the NC. These data were consistent with Konishi et al., (2009) from Japan. Thus, DM may have a role in the pathogenesis of chronic HCV hepatitis.

Other studies from japan reported that the risk of HCC in chronic HCV patients increases in overweight and obese patients compared to those underweight, hence increased BMI and obesity represent independent risk factors for HCC development in chronic HCV (Ohata et al., 2003; Ohki et al., 2008). In contrary, our data show no significant differences between the three tested groups regarding BMI, since the mean BMI in HCC, HCV and NC was (27.2±4.95, 28.4±4.1 and 28.6±6.6; respectively), and the mean body weight was (75.8±14.4, 79.3±13.3 and 76.7±16.8; respectively). Thus we assume that the increased serum levels of adiponecine, leptin and visfatin in HCC and HCV patients are possibly due to mechanisms other than obesity, and thus further studies are highly required to investigate the role of these adipokines in the pathogenesis of HCV, and the development of HCC. Furthermore, we observed a significant association between the serum levels of adiponectin, leptin and visfatin with the presence of cirrhosis in HCV patients. Meanwhile, there were no significant association with the BMI or a history of DM. These results confirm those of Sun et al., (2017) who reported increased serum visfatin level in HBV associated HCC in patients who have BMI<25 kg/m^2^. Therefore, further studies on a large number of patients are required to exactly determine the role of adipokines in HCC obese patients.

Similar to the classical cytokines, adipocytokines have many pro-inflammatory functions, as they increase the production of IL-6, TNF-α, and IL-12 (Moschen et al., 2007). overexpression of visfatin increases the activity of a number of signaling pathways that promote carcinogenesis e.g. NAD-dependent SIRTs, PI3K/ Akt, ERK1/2, and STAT3 (Bi and Che, 2010). 

According to our data, assessment of the serum levels of adiponecine, leptin and visfatin increase the diagnostic accuracy of HCC patients, leptin has the highest sensitivity (98.8%), and visfatin has the highest specificity and PPV (100%). However, these data are not consistent with Sun et al., (2017), who reported 45.76% sensitivity, and 74.79% specificity values of serum visfatin for the diagnosis of HCC.

We recommend using a combination of serum leptin and visfatin for the diagnosis and prediction of HCV associated HCC patients, as the sensitivity, specificity, PPV, NPV and accuracy were (100%, 96.6%, 93.4%, 100% and 97.4%; respectively).

In conclusion, serum levels of adiponecine, leptin and visfatin increased significantly in HCC and HCV infected patients compared to the normal control subjects. Moreover, these markers are significantly associated with the presence of cirrhosis in HCV patients, despite there were no significant differences in the BMI among patients’ groups. Therefore, another mechanism(s) may be responsible for the elevation of these adipokines in the HCV associated HCC other than obesity. The combination of serum leptin and visfatin could be used as potential biomarkers for early detection and diagnosis of patients with HCV associated HCC. Further studies are still required on larger number of patients to confirm the role of these markers in HCC, also in the other malignancies. 
